# 

**Published:** 1915-04-10

**Authors:** 


### CONTENTS.

PAGE

**Medical Baths for Invalid Soldiers** ... ... 23

**Institutions and the Drug Supply** ... ... 24

**Hospital and Institutional News** ... ... ... 25

THE HOSPITAL'S Special Number on "Transport"—The Overworking of Infirmary Medical Officers—Poor-Law Medical Officers and Clerical Work—Women as Sanatoria Medical Officers—St. Thomas's Hospital Hostel—The Theft of X-Ray Apparatus—Bristol Royal Infirmary as a Base Hospital—New President of the Royal College of Physicians—The Fourth Serbian Relief Unit—Queen Alexandra and the St. Katharine's Hospital Site—The Return to Work of Discharged Consumptives—The Evolution of Hospital Architecture—Anomalous Salaries in the Poor-Law Medical Service—An Officer's Memorial—Medical Pressure at Camberwell Infirmary—A Strike at a Hospital—Short Staffs and the Annual Holiday—A Presentation for War Services—A Mental Hospital Attendant Charged—The Princess Christian Hospital Train—Women and Hospitals After the War—The Late Lord Rothschild—This Week's Drug Market.

**Su gical Experiences** ... ... ... ... ... 31

The Septic Infection of Wounds.

**Hypnosis Produced in Battle** ... ... ... 32

A French Practitioner's Experiences.

**The Handicap of the First-Born** ... ... ... 33

The Small Family and Racial Progress.

**Four Remarkable Cases** ... ... ... ... 34

Genito-Urinary Sporotrichosis in New Zealand.

[*See over.*]

### CONTENTS—(*continued*).

| | PAGE |
|---|---|
| **Condensed and Dried Milk and Tuberculosis** ... | 35 |
| The Infectivity of Preserved Specimens. | |
| **The Evolution of the Modern Hospital** ... ... | 36 |
| VI.—The Performance of Operations. | |
| **Hospital Architecture and Construction** ... | 37 |
| The John Coupland Hospital, Gainsborough. | |
| **Buildings Contemplated and in Progress** ... | 38 |
| **Gifts and Bequests** ... ... ... ... ... | 38 |
| **Round the World** ... ... ... ... ... | 39 |
| Fellow-Workers and the Roll of Honour. | |
| **Practical Points in Diagnosis and Treatment**... | 40 |
| **The Military Cross for an Army Doctor** ... | 42 |
| **Pubiic Pharmacists' "Open Evening"** ... ... | 42 |
| **The Late Dr. W. Lewis Thomson**... ... ... | 42 |
| **Obituary** ... ... ... ... ... ... ... ... | 42 |
| **Doctors' Wills** ... ... ... ... ... ... ... | 42 |
| **Medical Appointments** ... ... ... ... ... | 44 |
| **Personalia** ... ... ... ... ... ... ... ... | 44 |
| **Institutional Needs** ... ... ... ... ... ... | 44 |
| **The Book Market** ... ... ... ... ... ... | 44 |
| **The Institutional Worker** ... ... (Suppl.) | 1 |
| III.—The Steward. | |
| Questions for April. | |
| Enquiries and Answers. | |
| War Matters. | |
| Miscellaneous. | |

